# Healthy and Sustainable Food Shopping: A Survey of Intentions and Motivations

**DOI:** 10.3389/fnut.2022.742614

**Published:** 2022-03-02

**Authors:** Julia Blanke, Joël Billieux, Claus Vögele

**Affiliations:** ^1^Department of Behavioural and Cognitive Sciences, University of Luxembourg, Esch-sur-Alzette, Luxembourg; ^2^People Behaviour & Technology Integration Research Group, Munster Technological University, Cork, Ireland; ^3^Institute of Psychology, University of Lausanne, Lausanne, Switzerland

**Keywords:** health, sustainability, grocery shopping, behavioral intention, motivation, theory of planned behavior, self-determination theory

## Abstract

**Objectives:**

To determine the relationship between sustainable and healthy food shopping behavior comparing general motivation with the immediate intention to act.

**Method:**

We conducted an online survey of 144 staff at the Cork Institute of Technology, Ireland, using a questionnaire based on the Theory of Planned Behavior and the Self-Determination Theory to compute the Behavioral Intention score and the Relative Autonomy Index in relation to healthy and sustainable grocery shopping.

**Results:**

The intention to shop healthy food was higher (*p* < 0.001, Cohen's d = 0.56) than the intention to shop in a sustainable way. A significant intention-action gap was observed for both healthy (*p* < 0.001, Cohen's d = 0.97) and sustainable grocery shopping (*p* < 0.001, Cohen's d = 1.78). While there was a significant correlation (*p* < 0.001) between the longer-term motivations to act in a healthy and sustainable way, this association was not significant (*p* = 0.16) for the more short-term Behavioral Intention scores.

**Conclusion and Implications:**

Health was identified as a more important driver for dietary behavior compared to sustainability. While longer-term motivation shows a stronger correlation between healthy and sustainable grocery shopping, short-term intentions do not follow this pattern as strongly. A significant intention-action gap exists for both, which is stronger for sustainability than for health.

## Introduction

Sustainability and public health are both challenges for current global dietary trends (1), and by proxy food shopping behavior. Both on the international (2) and national levels [e.g., (3)] food-based dietary guidelines (FBDGs) are developed; however, most do not consider sustainability explicitly (4) and predominantly focus on health-related recommendations (5) targeting environmental sustainability by means of alignment with healthy dietary patterns (6). A recent literature review by Biasini et al. (7) concluded that the health dimension of sustainability dominates whereas environmental dimensions have been poorly investigated. While healthy and sustainable diets show a significant overlap (8) and the co-benefit between health and sustainability should be promoted (9), differences between the two topics have to be acknowledged (10) and should be addressed independently if necessary.

FBDGs are developed at the policy level with the primary goal of improving population health and reducing environmental impacts. At the same time a shift in consumer behavior (11) is driving changes on the supply side of the food industry with sustainability marketing (12, 13) being increasingly implemented. Sustainability information in shopping situations (14) is recognized as relevant for designing choice architectures (15), i.e. how products are presented to consumers, and for shaping consumer decision making practices (16, 17). For instance, Canio and Martinelli, (18) found that consumers are willing to pay a premium price for sustainable EU quality label foods, although an intention-action gap has been observed.

At the individual level behavioral factors need to be understood to shift grocery shopping behavior toward more sustainable options (19). Because of the close relationship between health and sustainability (20), research has focused on the health aspects of sustainable diets (21) as well as the sustainability aspects of healthy diets (22). An explicit distinction between the two topics with respect to individual behavioral factors has been made in the qualitative study by Hoek et al. (23), who found that health is still a more important driving motivator for choices of personal diets.

In this work we aim to address the intention and motivation of people with respect to health and sustainability with a focus on shopping behavior as an essential part of a healthy and sustainable diet. A distinction is made between the long-term motivation, which refers to general beliefs and tendencies toward the topic, and the more short-term intention to act, which is the condition shortly before showing a behavior in question focusing on a single goal. The aim of this work is to explore all the mutual relationships between these two concepts with regards to both health and sustainability. We use a theory-based approach to assess and evaluate variables relating to intention and motivation based on the Theory of Planned Behavior (TPB) (24, 25) and the Self-Determination Theory (SDT) (26) to answer the following research questions:

a. Are the intentions to buy healthy or sustainable groceries at similar levels, and if not, in what way do they differ?
b. Are the longer-term motivations to buy healthy or sustainable groceries at similar levels, and if not, in what way do they differ?
c. Is healthy grocery shopping behavior considered more likely than sustainable grocery shopping behavior or vice versa, and how does this compare to the intention?
d. Are there intention-action gaps for healthy and sustainable grocery shopping behavior, and if so, are there differences in their respective size?
e. Does a higher general motivation to shop healthily also imply a higher motivation to shop sustainably and vice versa?
f. Does a higher immediate intention to shop healthily also imply a higher intention to shop sustainably and vice versa?
g. Does longer-term motivation translate into short-term intentions in the same way for healthy and for sustainable grocery shopping?

While the two topics are expected to be related in how people make their grocery shopping decisions (23), in the following we aim to analyse the differences between long-term motivation and short-term intention and behavior with respect to healthy and sustainable grocery shopping behavior. Other aspects affecting food choice such as price, taste, quality, and convenience (13) or personal dietary patterns (e.g., omnivore, flexitarian, vegetarian, etc.) are beyond the scope of this paper. Physiological and emotional aspects, like hunger or feeling sad, angry or happy, were not considered, although they most likely have an impact on grocery shopping behavior as well (27). The presented study was conducted in Ireland, where sustainability is not yet part of current FBDGs (5).

We used the definition of a healthy diet provided by the World Health Organization to be understood as balanced and based on plenty of vegetables and fruit, reduced fat (particularly the wrong type), and limited intake of sugars and salt (2). Defining an environmentally sustainable diet is more complex (28). The Food and Agriculture Organization of the United Nations (29) defines sustainable diets as having low environmental impact, contributing to food and nutrition security, and delivering a healthy life for present and future generations. Instead of using this very comprehensive definition, encompassing many aspects that are difficult to quantify and measure, many studies focus on greenhouse gas emissions (28, 30). In this paper we defined sustainability as reduced ecological footprint related to carbon emission, water and energy use as well as less animal- and more plant-based diets and seasonal products (13, 23). Both definitions were provided to the study participants (see Supplementary Material).

## Method

### Participants and Recruitment

The study was advertised at the Cork Institute of Technology (CIT), a third level education institution in Ireland. CIT was chosen to represent a regional hub catering for the full range of post-secondary education ranging from level 6 to 10 of the European Qualifications Framework. An email was sent to a general email distribution list used by staff to communicate and advertise topics of general interest, where some may not be work related. Reaching 1,425 potential participants comprising all academic, administrative, and services staff of the institute the email advertised the study and provided a link to participate in the online survey (see Supplementary Material). Data was collected in April/May 2019. This date was chosen after the Easter Break and during the teaching semester to ensure maximum staff availability. The inclusion criteria were being a member of staff at CIT at the time of the survey and having a CIT email address. The survey included an initial information section on the purpose of the study and the use of data (see Supplementary Material). Participation was voluntary with no incentives being given. Furthermore, the questionnaire was anonymous with no meta-data collected, which would have enabled identification of participants (e.g. IP addresses). The study was approved by the Ethics Review Panel of the University of Luxembourg and the Ethics Review Board of the Cork Institute of Technology.

### Inventories

The questionnaire was built based on the Theory of Planned Behavior (TPB) and the Self-Determination Theory (SDT), both providing guidance on how to develop suitable inventories. Both theories outline in detail how questions must be formulated and have been empirically validated in many previous studies [e.g., (7, 18, 31, 32)]. By following these exact layouts (see Supplementary Material) we tried to ensure the validity of the measured variables.

The TPB proposes the assessment of three distinct contributors: attitudes (A), subjective norms (SN), and perceived behavioral control (PBC), for which we developed questions accordingly to assess the strength of the relevant beliefs on a 5-point Likert scale to be aggregated into the Behavioral Intention (BI, see below) scores (24).

While the TPB is used to assess behavioral intention, the SDT provides a model and operationalisation for motivation. In accordance with the SDT we developed survey questions to assess the external regulation (R^Ext^), the introjected regulation (R^Intro^), i.e. avoidance of negative consequences, the identified regulation (R^Id^), i.e. acknowledgment of the importance of a goal, and the intrinsic regulation (R^Int^) to measure their respective strength on a 5-point Likert scale. Based on these, the type and strength of motivation is operationalised as the Relative Autonomy Index (RAI, see below) (26), with higher RAI scores representing higher intrinsic motivation, and lower RAI scores indicating extrinsic motivation.

These 7 parameters required for the TPB and the SDT can be assessed separately for both topics health as well as sustainability resulting in a total of 14 variables. The final online survey (see Supplementary Material) comprised a total of 8 sections out of which 4 where designed to evaluate the variables relating to the TPB and the SDT: section 3 assessed *A*_*H*_, *SN*_*H*_, and *PBC*_*H*_ with respect to health, section 4 assessed $R_{H}^{Ext}$, $R_{H}^{Intro}$, $R_{H}^{Id}$, and $R_{H}^{Int}$ with respect to health, section 6 assessed *A*_*S*_, *SN*_*S*_, and *PBC*_*S*_ with respect to sustainability, and section 7 assessed $R_{S}^{Ext}$, $R_{S}^{Intro}$, $R_{S}^{Id}$, and $R_{S}^{Int}$ with respect to sustainability. The final variables were calculated as the mean of the respective answers and are therefore all on a scale between −2 and +2.

In addition to these, 4 more variables were assessed: self-reported intention (I_H_) and perceived actual behavior (B_H_) with respect to health (section 2) as well as with respect to sustainability [I_S_, B_S_, section 5]. Again, all variables were assessed on a 5-point Likert scale.

Finally, demographic data (section 1) and a ranking of other factors (taste, price, quality etc.) relevant to grocery shopping behavior has been included in the survey (section 8).

### Data Aggregation

**Figure 1:**
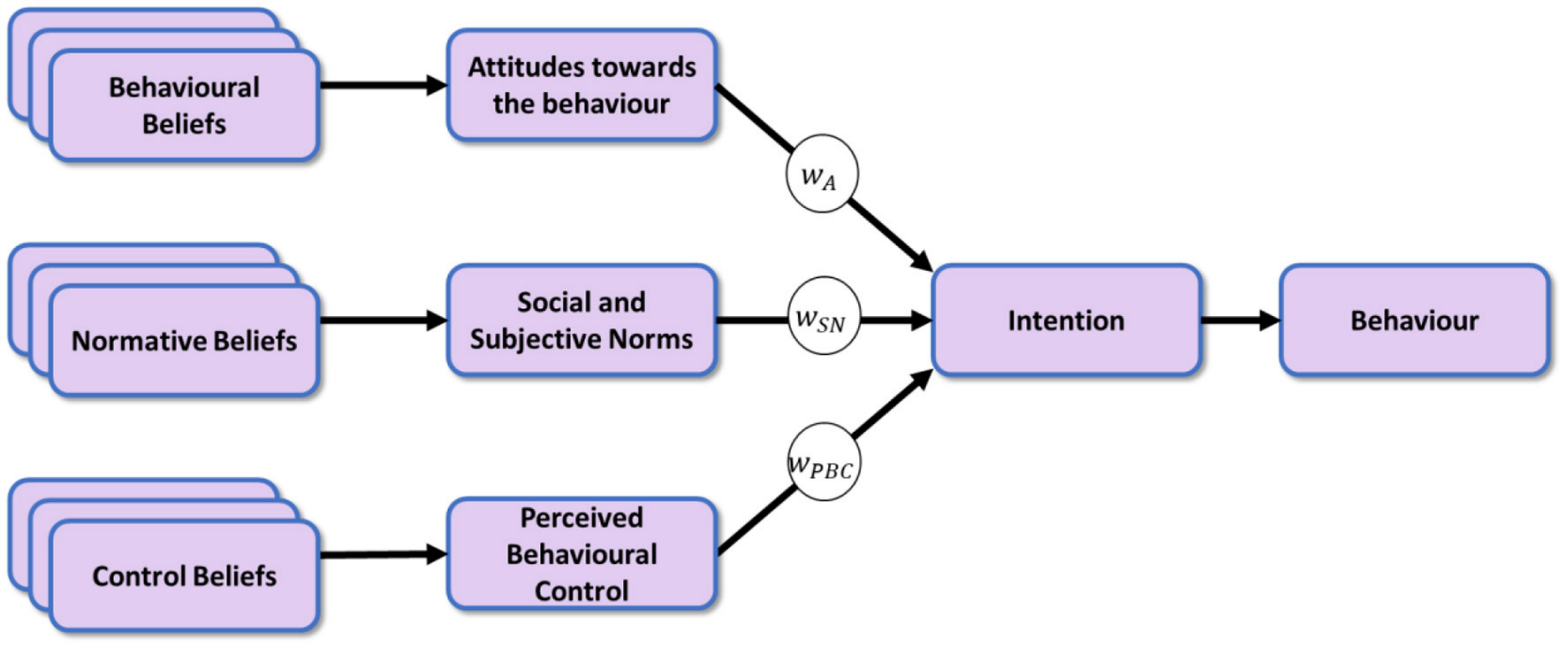
The theory of planned behavior (TPB).

The TPB proposes the Behavioral Intention score (BI) to measure the probability of an individual to act toward a given objective such as healthy or sustainable grocery shopping. The BI is calculated as the weighted sum (see Figure 1) of the attitude toward the behavior A, the subjective norm SN, and the perceived behavioral control PBC (24).


$$\begin{array}{l} BI=w_{A}A+w_{SN}SN+w_{PBC}PBC \end{array}$$

The weight factors for calculating the behavioral intentions BI_H_ and BI_s_ with respect to healthy and sustainable behavior have been determined by maximizing the respective Pearson correlations between BI_H_ and the self-reported intention I_H_ to r_(142)_ = 0.41, *p* < 0.001 (α_*BH*_=0.032), and between BI_s_ and I_s_ to r_(142)_ = 0.55, *p* < 0.001 (α_*BH*_=0.024). The resulting normalized weights for the computation of BI_H_ were determined as *w*_*A*_ = 1.4, *w*_*SN*_ = 1.0, *w*_*PBC*_ = 0.5, and the weights for computing BI_S_ were *w*_*A*_ = 1.6, *w*_*SN*_ = 0.5, *w*_*PBC*_ = 0.5. With A, SN, PBC measured on a scale between −2 and +2 the resulting range of potential BI values is therefore normalized to fall between −6 and + 6.

The data from the TPB also provides means for building segments (personas) of different people enabling individualized profiles that can give an indication what kind of support or feedback is needed to keep people motivated. This categorization is based on thresholding the three variables *A*, *SN*, and *PBC* resulting in 8 different outcomes depending on the combination of the three being either positive or negative (see Table 1).

**Table 1:** Possible persona segments derived from the TPB.

**Persona**	**Attitude**	**Social pressure awareness**	**Perceived behavioral control**
1	Positive	High	High
2	Positive	Low	High
3	Positive	High	Low
4	Positive	Low	Low
5	Negative	High	High
6	Negative	Low	High
7	Negative	High	Low
8	Negative	Low	Low

**Figure 2:**
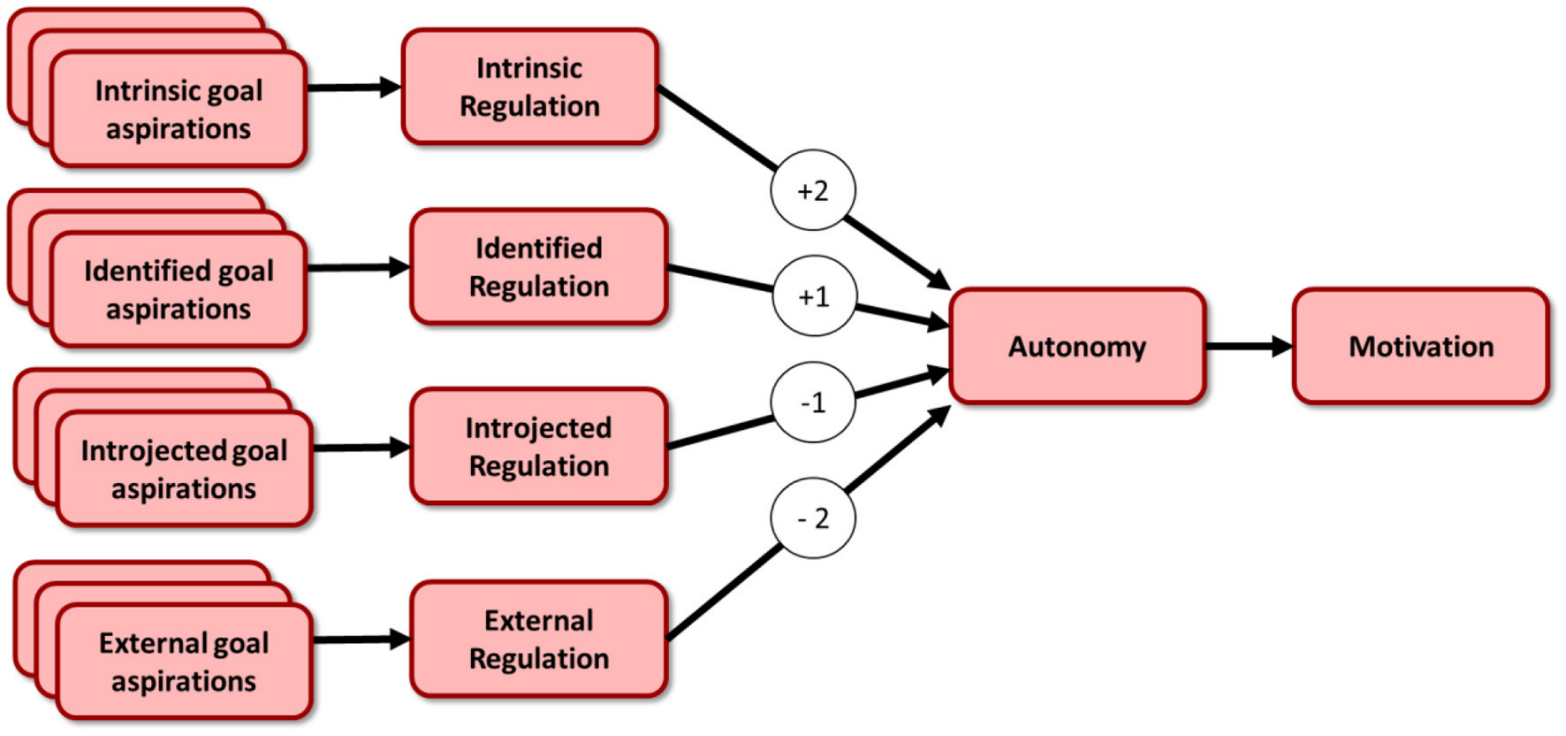
The self-determination theory (SDT).

To calculate the RAI the SDT proposes to assess four distinct contributors: the external, introject, identified, and the intrinsic regulation. Similar to the TPB these measures are aggregated into the Relative Autonomy Index (RAI) defined as the weighted sum (see Figure 2) of the respective answers summed up, with the weight factors in this case pre-defined according to (26). With the regulations measured on a scale ranging from −2 to +2 the RAI values therefore fall between −12 and +12.


$$\begin{array}{l} RAI=2R^{Int}+R^{Id}-R^{Intro}-{2R}^{Ext} \end{array}$$

The BI score and the RAI can be calculated for both health and sustainability separately. We also evaluate how these indicators compare with self-report behavior and intention, which have been separately surveyed through the questionnaire on a 5-point Likert scale. In summary, the parameters listed in Table 2 were assessed and computed to determine the relationship between sustainable and healthy food shopping behavior and to compare the general motivation with the more immediate intention to act.

**Table 2:** Abbreviations for the variables (including means and standard deviations).

**Parameter**	**Abbreviation**	**m**	**SD**
Self-reported intention to act healthily	I_H_	1.38	0.69
Self-reported healthy behavior	B_H_	0.4	0.86
Self-reported intention to act sustainable	I_S_	0.77	0.93
Self-reported sustainable behavior	B_S_	−0.37	1.03
Behavioral intention to act healthily (TPB)	BI_H_	2.33	1.45
Behavioral intention to act sustainable (TPB)	BI_S_	0.92	1.28
Relative autonomy index with regards to health (SDT)	RAI_H_	2.12	2.48
Relative autonomy index with regards to sustainability (SDT)	RAI_S_	2.04	2.02

### Data Analytic Procedure

In the following we are addressing the 7 research questions (a–g) as posed in the introduction based on the derived variables. To that end the statistical analyses conducted included *t-*tests between I_H_ and I_S_ to answer question (a), between RAI_H_ and RAI_S_ to answer question (b), between B_H_ and B_S_ to answer question (c), and between I_H_ and B_H_- as well as between I_S_ and B_S_ to answer question (d). We also report the corresponding effect sizes (Cohen's d). Furthermore, Pearson correlations were calculated between RAI_H_ and RAI_S_ to answer question (e), between BI_H_ and BI_S_ to answer question (f), and between RAI_H_ and BI_H_ as well as between RAI_S_ and BI_S_ to answer question (g). In order to compensate for multiple comparisons, we applied the Benjamini-Hochberg correction (33) and used stricter thresholds for statistical significance of between α_*BH*_ = 0.003 to α_*BH*_ = 0.05 as indicated where appropriate.

## Results

Although 176 respondents opened our survey hyperlink, a total of 144 (*n* = 144) provided answers to all the relevant sections of the questionnaire relating to the TPB and SDT for a 10% response rate. Of these 53% were female, 31% male and 16% did not answer this question. One third of participants (33%) were between 45–54 years old, 27% were between 35–44, 17% were between 55–64, and 11% were between 25–34 years old. The age groups with the smallest participant rates were the 18–24 year-olds and the 65+ cohort, with a percentage of 2 and 3% respectively. In total 6% of participants chose not to disclose their age. The distribution of education level shows that the largest group (48%) was represented by people with a level 9 (Master's) degree followed by 21% holding a level 8 (Bachelor's) degree and 15% with a PhD. Only 16% of participants had a level 7 degree or lower, as would be expected amongst staff of a third level education institution. The datasets presented in this study can be found in the Open Science Foundation repository here: https://osf.io/usp6f/.

### Self-Reported Intentions and Behavior

The distribution of participants with respect to self-reported intention showed a mean of m = 1.38 and a standard deviation of SD = 0.69 for health (I_H_) and a mean of m = 0.77 and a standard deviation of SD = 0.93 for sustainability (I_S_). A significant Pearson correlation, *r*_(142)_ = 0.35, *p* < 0.001 (α_*BH*_=0.041), between the two was observed. Furthermore, there was a significant difference between the self-reported importance of health and sustainability, t_(143)_ = 7.84, *p* < 0.001 (α_*BH*_=0.021), with health being considered more important than sustainability by participants, Cohen's d = 0.56 [question (a)].

Similarly, self-reported behavior showed a mean of m = 0.4 and a standard deviation of SD = 0.86 with regards to health (B-_H_) and a mean of m = −0.37 and a standard deviation of SD = 1.03 with regards to sustainability (B-_S_). Out of the 144 participants 6 answered “Don't know” in this category and were excluded from the analysis. A significant Pearson correlation, *r*_(136)_ = 0.4, *p* < 0.001 (α_*BH*_=0.035), between self-reported behavior of health and sustainability was shown. Again, there was a significant difference between the self-reported behavior with regards to health and sustainability, t_(137)_ = 8.22, *p* < 0.001 (α_*BH*_=0.015), with health being considered more important than sustainability by participants, Cohen's d = 1.95 [question (c)].

Comparing this to the Cohen's d for self-reported intention (see above), the difference between health and sustainability is much larger in self-reported behavior than in self-reported intention.

### Self-Reported Intention-Action Gap

When comparing self-reported health intention and self-reported health behavior a significant Pearson correlation of r_(142)_ = 0.64, *p* < 0.001 (α_*BH*_=0.009), was observed. Similarly, the survey also showed a statistically significant correlation, *r*_(136)_ = 0.57, *p* < 0.001 (α_*BH*_=0.018), between self-reported sustainability intention and self-reported sustainability behavior.

Both show a gap between self-reported intention and self-reported behavior. The difference between self-reported intention and self-reported behavior was significant with regards to health, t_(143)_ = 17.42, *p* < 0.001 (α_*BH*_=0.003), as well as sustainability, t_(137)_ = 14.7, *p* < 0.001 (α_*BH*_=0.006). The self-reported intention was larger in both cases; however, while we observed a Cohen's d = 0.97 for health, the difference was even larger for sustainability with a Cohen's d of 1.78 [question (d)].

### The Behavioral Intention

The following evaluation is based on the Theory of Planned Behavior (24) as outlined above. The behavioral intention score toward healthy (BI_H_) and sustainable grocery shopping behavior (BI_S_) was calculated. It showed a mean and standard deviation of m = 2.33 and SD = 1.45 for BI_H_ and m = 0.92 and SD = 1.28 for BI-_S_. The Behavioral Intention to act healthily was significantly higher than the Behavioral Intention to act sustainably, t_(143)_ = 9.29, *p* < 0.001 (α_*BH*_=0.012), Cohen's d = 1.04. No significant correlation was observed, *r*_(142)_ = 0.12, *p* = 0.16 (α_*BH*_ = 0.047) [question (f)].

### Segments/Personas

The TPB also allows for the analysis of different segments of people (personas) according to their respective beliefs. In the present study the largest group consisted of participants (16%) who report positive attitudes and high social pressure awareness toward both health and sustainability, but differ in perceived behavioral control, which was higher for health (persona 1; cf. Table 1) than for sustainability (persona 3; cf. Table 1). The second largest group (11.1%) showed positive attitudes, high social pressure awareness, and low perceived behavioral control (persona 3, cf. Table 1) with respect to both health and sustainability. The difference between health and sustainability can be attributed to differences in perceived behavioral control, with 87.5% of respondents showing a low value in this category (personas 3,4,7, and 8; cf. Table 1) with respect to sustainability compared to 56.3% showing the same in the health category. This difference is significant as indicated by a Wilcoxon signed rank test resulting in W = 1,070, *p* < 0.001 (α_*BH*_=0.038).

### The Relative Autonomy Index

This analysis was based on the Self-Determination Theory (26). The Relative Autonomy Index with respect to healthy (RAI_H_) and sustainable (RAI_S_) grocery shopping behavior showed a mean and standard deviation of m = 2.12 and SD = 2.48 for RAI_H_ and m = 2.04 and SD = 2.02 for RAI_S_. There was no discernible difference between the Relative Autonomy Index with respect to healthy eating behavior and the Relative Autonomy Index with regard to sustainable eating, t_(143)_ = 0.39, *p* = 0.7 (α_*BH*_ = 0.05), Cohen's d = 0.03. Unlike the Behavioral Intention (BI) discussed above, the motivational type as measured by the RAI, therefore, indicated similar levels of general interest in both topics. Supporting this result, a correlation of *r*_(142)_ = 0.49, *p* < 0.001 (α_*BH*_ = 0.026), between the two dimensions was observed, indicating again that motivational types do not depend on the respective subject area and that, therefore, incentives are working similarly with respect to both healthy and sustainable behavior [questions (b) and (e)].

Finally, we examined the relationship between Behavioral Intention (BI) and the Relative Autonomy Index (RAI). A significant correlation, r_(142)_ = 0.44, *p* < 0.001 (α_*BH*_ = 0.029), between the two with respect to healthy behavior was observed. The same was true for sustainable grocery shopping behavior, r_(142)_ = 0.23, *p* < 0.01 (α_*BH*_ = 0.044), although the correlation coefficient was somewhat smaller [question (g)].

All statistical results in relation to the addressed research questions as introduced in the introduction are summarized again in Table 3.

**Table 3:** Summary of results.

**Question**			**d**	**r**	**p**	**α_BH_**	**reject H_**0**_**
(a)	I_H_	I_S_	0.56		<0.001	0.021	Yes
(b)	RAI_H_	RAI_S_	0.03		0.7	0.05	No
(c)	B_H_	B_S_	1.95		<0.001	0.015	Yes
(d)	I_H_	B_H_	0.97		<0.001	0.003	Yes
	I_S_	B_S_	1.78		<0.001	0.006	Yes
(e)	RAI_H_	RAI_S_		0.49	<0.001	0.026	Yes
(f)	BI_H_	BI_S_		0.12	0.16	0.047	No
(g)	BI_H_	RAI_H_		0.44	<0.001	0.029	Yes
	BI_S_	RAI_S_		0.23	<0.01	0.044	Yes

## Discussion

The main goal of this study was to evaluate the relationship between health and sustainability in relation to dietary behavior and grocery shopping with a focus on intention, motivation, and behavior. To this end we conducted a survey based on the Theory of Planned Behavior (TPB) and Self-Determination Theory (SDT) in 144 participants. While most previous studies based on the TPB focused on intentions in relation to healthy dietary behavior, thus far very little work has been carried out with respect to sustainability, and the relationship between health and sustainability [see (7) for a systematic review]. To our knowledge, neither a quantitative comparison between intentions nor a comparison with respect to motivational regulation as defined by the SDT between healthy and sustainable dietary behavior has been conducted previously.

First, we evaluated self-reported intentions and behaviors, both in relation to a healthy lifestyle as well as in relation to sustainability (I_H_, B_H_, I_S_, B_S_). We then determined the Behavioral Intention scores as defined by the TPB, and Relative Autonomy Indices as defined by the SDT for both healthy and sustainable grocery shopping behavior (BI_H_, BI_S_, RAI_H_, RAI_S_). The weights for the TPB were calibrated by maximizing the correlation between I_S_ and BI_S_ as well as between I_H_ and BI_H_. Based on these 8 parameters, which all aim at explaining intentions and motivations with respect to health and sustainability, we derived the following conclusions.

The intention of participants to follow a healthy diet I_H_ is significantly higher (Cohen's d = 0.56) than the intention to buy sustainable groceries I_S_ (a). While this is consistent with the findings of (23), we found this not to be the case for longer-term motivation (b), though, where only a marginally significant difference between the RAI_H_ and the RAI_S_ has been observed. This indicates that there is a gap between long term motivation and short-term intention. While the motivations for both health and sustainable grocery shopping are at similar levels, this does not translate into the short-term intention to act in the same way. This is consistent with the finding that self-reported behavior to shop healthily B_H_ is more likely than to shop sustainably B_S_ (c), with the difference between the two (Cohen's d = 1.95) exceeding the difference for the intention. The gap seems to widen the more concrete the behavior becomes. Both show a significant gap (d) between intentions (I_H_, I_S_) and behaviors (B_H_, B_S_), with the gap for sustainability being even larger (Cohen's d = 1.78) than for health (Cohen's d = 0.97). It seems to be more difficult to translate an intention to shop sustainable groceries into action than it is to do the same for healthy products. This is consistent with the finding, that perceived behavioral control is a more dominant issue for sustainable grocery shopping. Longer-term motivations for health (RAI_H_) and sustainability (RAI_S_) are significantly correlated (e); however, this is not true for short-term intentions (BI_H_, BI_S_) (f). Again, this shows the difference between long term motivation and short-term intention, where the general motivation toward one topic predicts the general motivation toward the other, but the same cannot be said for the immediate intention to act. Nevertheless, looking at the correlation between motivation and intention (g), we found that for both health (RAI_H_, BI_H_) as well as for sustainability (RAI_S_, BI_S_), the longer-term motivational predisposition is significantly associated with the short-term intention to execute the behavior in question. Again, the correlation was weaker for sustainability than it was for health (r_H(142)_ = 0.44 > r_S(142)_ = 0.23).

Verain et al. (34) emphasize the importance of building segments for influencing healthy and sustainable diet intentions. Here we proposed a TPB-derived methodology for the categorization of participants based on the explicit assessment of attitudes, subjective norms, and perceived behavioral control. We found that the drivers for healthy behavior and the drivers for sustainable behavior are not necessarily the same for a significant proportion of participants, with the main difference being that a lower perceived behavioral control exists with regard to sustainable grocery shopping. This is consistent with findings that self-efficacy is amongst the main drivers for the uptake of a healthy and sustainable diet (19) and that more support is required to empower people to act sustainably (35).

On average, we observed higher intentions for healthy behavior than for sustainable behavior, which is consistent with previous research (23); we also found that motivation does not translate as well into intention and action for sustainable than for healthy grocery shopping. Negative intentions with regards to healthy behavior were not reported while the spectrum of answers on the sustainability scales was broader. This indicates that health aspects are considered more important than sustainability aspects concerning grocery shopping behavior. Awareness of sustainability issues is lower than awareness concerning healthy eating behavior. This is also consistent with the finding that the self-reported importance of sustainability ranked lower than the importance of health concerning grocery shopping.

## Limitations

The main limitation of this study is that the evaluation was only executed in one country amongst staff at Cork Institute of Technology, Ireland. The self-reported profile of participants has been leaning toward the higher end of education level compared to the population average, and the median age was over 45. This limits the generalization of the current findings. An analysis of gender, age, or education on the Behavioral Intention or the Relative Autonomy Index showed no significant differences or relationship between these categories and the two indicators. For example, Behavioral Intention did not differ between male and female participants.

A second limitation is the low response rate of 10%. The survey was advertised on a general mailing list to all staff, with 144 of the 1,425 recipients participating in the survey. While a non-response bias cannot be ruled out (36), its negative impact on the validity of results would have been reduced by participation being driven by goodwill and not being affected by perceived gains (37). Nevertheless, as with all voluntary questionnaires a selection bias toward more positive attitudes regarding the subject area is a possibility. Duplicate responses, forwarding the link to others, or providing wrong answers can also not be ruled out, although we believe this to be unlikely. The survey was anonymous and without any incentives, which might have contributed to a drop-out toward the end of the questionnaire, reducing the usable data sample from the initial number of 176 to 144 participants. Other aspects limiting this study are that mainly women took part, and that young people between 18 and 24 were under-represented.

The definitions of healthy and sustainable diets given to the study participants were focusing on nutritional values and the carbon footprint of the food products in general, even if this is not reflective of the full complexity of the two topics. The study also did not go into any detail with respect to a particular shopping behavior (e.g. online vs. brick-and-mortar) or particular marketing instruments (e.g. eco-labeling). The results are therefore based on the self-reflection and general understanding of participants, and do not distinguish between particular diseases (e.g. cardio-vascular, diabetes, etc.) or environmental factors (e.g. packaging, processing, farming practices, etc.). We assume that participants understood the surveyed concepts based on the explanations provided. Although the validity of this assumption has not been tested, the higher-than-average education level of participants could mitigate this risk. However, it cannot be ruled out that this assumption has an impact on the results.

## Conclusion

Health is still the major driver concerning dietary behavior, and this fact is evident across the presented evaluations. Overall, the intention to act healthily is higher than the intention to act sustainably.

In terms of long-term motivation, a stronger correlation between health and sustainability can be observed compared to the short-term intention, where no such association was found. Also, the long-term motivation correlates more strongly with the short-term intention for healthy dietary behavior than for sustainability. Altogether, this indicates that the translation of long-term motivation into actual behavior is less likely for sustainability than it is for health.

Furthermore, a significant intention-action gap exists for both healthy and sustainable grocery shopping behavior. Looking at the persona segmentation, the main difference between health and sustainability occurs with respect to perceived behavioral control indicating that suitable guidance with the aim to overcome this issue could be a successful approach to improving sustainable behavior. Improving overall sustainable outcomes in relation to grocery shopping behavior could, therefore, benefit from personalized and individual measures tailored toward improving this aspect in particular.

## Data Availability Statement

The datasets presented in this study can be found in the following Open Science Foundation repository: https://osf.io/usp6f/.

## Ethics Statement

The studies involving human participants were reviewed and approved by Ethics Review Panel of the University of Luxembourg and the Ethics Review Board of the Cork Institute of Technology. Written informed consent for participation was not required for this study in accordance with the national legislation and the institutional requirements.

## Author Contributions

JBl conceived of the idea, designed and conducted the research, data analysis, and wrote the first draft of the manuscript. JBi and CV contributed to the design and analyses and structuring and writing the manuscript. All authors contributed to manuscript revision, read, and approved the submitted version.

## Conflict of Interest

The authors declare that the research was conducted in the absence of any commercial or financial relationships that could be construed as a potential conflict of interest.

## Publisher's Note

All claims expressed in this article are solely those of the authors and do not necessarily represent those of their affiliated organizations, or those of the publisher, the editors and the reviewers. Any product that may be evaluated in this article, or claim that may be made by its manufacturer, is not guaranteed or endorsed by the publisher.
