# Supplementary material for: Healthy and Sustainable Food Shopping: A Survey of Intentions and Motivations
Source: Front Nutr. 2022 Mar 2;9:742614. doi: 10.3389/fnut.2022.742614 (PMC8924458; doi:10.3389/fnut.2022.742614)
Supplement: Supplementary file 1 [file Data_Sheet_1.pdf]

Blanke, J., Billieux, J., & Vögele, C. (2022). Healthy and sustainable food shopping – A Survey of Intentions and Motivations. *Frontiers in Nutrition*, doi:10.3389/fnut.2022.742614.

Supplementary material: Questionnaire

What is this questionnaire about?
This questionnaire is about evaluating and accessing your healthy and sustainable food shopping behaviour. Furthermore, will we ask you about your motivation and intention to keep a healthy diet and buy sustainable food products.
Who can participate and how long will it take?
Everybody who is over 18 can participate. The questionnaire takes about 10-15min.
What happens with the collected data?
The questionnaire is anonymous, and the collected data are only used for research purposes. This research project was approved by the Research Ethics Committee at the Cork Institute of Technology and the ethical commission of the University of Luxembourg (ERP 18-078) (IMpreSS).
What happens if I want to stop in the middle of the questionnaire?
Your participation is on a voluntary base. You can stop at any time by closing the browser window without any personal disadvantages.
If you have any questions or concerns, please contact: Julia.Blanke@cit.ie

Section 1 – Demographics

1. What is your age:
    - 18-24
    - 25-34
    - 35-44
    - 45-54
    - 55-64
    - Over 65

2. What is your gender:
    - Male
    - Female
    - Other

3. What is your highest education level achieved?
    - Junior Certificate
    - Leaving Certificate
    - Higher Certificate
    - Ordinary Bachelor’s degree
    - Honours Bachelor’s degree or Higher diploma
    - Master’s degree
    - PhD degree
    - Other

Section 2 – Self-reported intention and behaviour (health)

In this section we will ask you about your healthy diet (healthy diet is understood as balanced and based on plenty of vegetables and fruit, reduced fat (particularly the wrong type), and limited intake of sugars and salt) [for further information you can look at: http://www.who.int/news-room/fact-sheets/detail/healthy-diet].

4. How important is a healthy diet for you personally?
    - Very important
    - Important
    - Neutral
    - Less important
    - Not important at all

5. In general, how healthy is your overall diet? Would you say . . .
    - Excellent
    - Very good
    - Good
    - Fair
    - Poor
    - Don't know

Section 3 – Behavioural Intention (health)

In this section we will ask you about your intentions regarding a healthy diet.

6. Sticking to a healthy diet is reasonable *(Attitude)*
    - Strongly disagree
    - Disagree
    - Neutral
    - Agree
    - Strongly agree

7. People who are important to me think that a healthy diet is overrated *(Subjective Norms, reversed question)*
    - Strongly disagree
    - Disagree
    - Neutral
    - Agree
    - Strongly agree

8. It is easy to stick to a healthy diet *(Perceived Behavioural Control)*
    - Strongly disagree
    - Disagree
    - Neutral
    - Agree
    - Strongly agree

9. Adhering to a healthy diet is a waste of time *(Attitude, reversed question)*
    - Strongly disagree
    - Disagree
    - Neutral
    - Agree
    - Strongly agree

10. Most of my friends are sticking to a healthy diet *(Subjective Norms)*
    - Strongly disagree
    - Disagree
    - Neutral
    - Agree
    - Strongly agree

11. To adhere to a healthy diet is difficult *(Perceived Behavioural Control, reversed question)*
    - Strongly disagree
    - Disagree
    - Neutral
    - Agree
    - Strongly agree

12. It is important to me that food I usually eat is healthy *(Attitude)*
    - Strongly disagree
    - Disagree
    - Neutral
    - Agree
    - Strongly agree

Section 4 – Relative Autonomy Index (health)

In this section we will ask you about your motivation staying on a healthy diet.
There are a variety of reasons why people trying to stay on a healthy diet. Please indicate how much you agree with the following statements:

13. Because I simply enjoy sticking to a healthy diet. *(Intrinsic Motivation)*
    - Strongly disagree
    - Disagree
    - Neutral
    - Agree
    - Strongly agree

14. Because a healthy diet is important and beneficial for my health and lifestyle. *(Identified Regulation)*
    - Strongly disagree
    - Disagree
    - Neutral

- Agree
- Strongly agree

15. Because I would feel bad about myself if I didn't adhere to a healthy diet. *(Introjected Regulation)*

- Strongly disagree
- Disagree
- Neutral
- Agree
- Strongly agree

16. Because it is fun and interesting to keep a healthy diet. *(Intrinsic Motivation)*

- Strongly disagree
- Disagree
- Neutral
- Agree
- Strongly agree

17. Because others like me better when I am on a healthy diet. *(External Regulation)*

- Strongly disagree
- Disagree
- Neutral
- Agree
- Strongly agree

18. Because I'd be afraid of the consequences if I'm not sticking to a healthy diet. *(Introjected Regulation)*

- Strongly disagree
- Disagree
- Neutral
- Agree
- Strongly agree

19. Because it helps my image keeping up with a healthy diet. *(External Regulation)*

- Strongly disagree
- Disagree
- Neutral
- Agree
- Strongly agree

20. Because it is personally important to me to have a healthy diet. *(Identified Regulation)*

- Strongly disagree
- Disagree
- Neutral
- Agree
- Strongly agree

21. Because I feel pressured to adhere to a healthy diet. *(Introjected Regulation)*
    - Strongly disagree
    - Disagree
    - Neutral
    - Agree
    - Strongly agree

22. Because I have a strong value for being healthy. *(Identified Regulation)*
    - Strongly disagree
    - Disagree
    - Neutral
    - Agree
    - Strongly agree

23. For the pleasure of discovering and mastering new things I try to stay on a healthy diet. *(Intrinsic Motivation)*
    - Strongly disagree
    - Disagree
    - Neutral
    - Agree
    - Strongly agree

24. Because I want others to see me as someone, who takes care of him-/herself. *(External Regulation)*
    - Strongly disagree
    - Disagree
    - Neutral
    - Agree
    - Strongly agree

Section 5 – Self-reported intention and behaviour (sustainability)

In this section we will ask you about your sustainable shopping behaviour (sustainability is understood as reduction of the ecological footprint related to carbon emission, water and energy use as well as less animal-based and more plant-based diets and seasonal products).

25. How important is sustainability for you when you go grocery shopping?
    - Very important
    - Important
    - Neutral
    - little important
    - Not important at all

26. In general, how sustainable is your overall grocery shopping behaviour? Would you say . . .
    - Excellent
    - Very good
    - Good

- Fair
- Poor
- Don’t know

Section 6 – Behavioural Intention (sustainability)

In this section we will ask you about your intentions to buy sustainable food products.

27. Buying sustainable food items is reasonable *(Attitude)*
    - Strongly disagree
    - Disagree
    - Neutral
    - Agree
    - Strongly agree

28. People who are important to me think that sustainability is overrated *(Subjective Norms, reversed question)*
    - Strongly disagree
    - Disagree
    - Neutral
    - Agree
    - Strongly agree

29. It is easy to buy sustainable food products *(Perceived Behavioural Control)*
    - Strongly disagree
    - Disagree
    - Neutral
    - Agree
    - Strongly agree

30. Figuring out which products are sustainable is a waste of time *(Attitude, reversed question)*
    - Strongly disagree
    - Disagree
    - Neutral
    - Agree
    - Strongly agree

31. Most of my friends are buying sustainable food products *(Subjective Norms)*
    - Strongly disagree
    - Disagree
    - Neutral
    - Agree
    - Strongly agree

32. To buy sustainable food products is not always easy *(Perceived Behavioural Control, reversed question)*
    - Strongly disagree

- Disagree
- Neutral
- Agree
- Strongly agree

33. It is important to me that food I usually buy is sustainable *(Attitude)*
    - Strongly disagree
    - Disagree
    - Neutral
    - Agree
    - Strongly agree

Section 7 – Relative Autonomy Index (sustainability)

In this section we will ask you about your motivation buying sustainable food.
There are a variety of reasons why people buy sustainable food. Please indicate how much you agree with the following statements:

34. Because I simply enjoy sustainable food. *(Intrinsic Motivation)*
    - Strongly disagree
    - Disagree
    - Neutral
    - Agree
    - Strongly agree
35. Because a sustainable food is important and beneficial for my health and the environment. *(Identified Regulation)*
    - Strongly disagree
    - Disagree
    - Neutral
    - Agree
    - Strongly agree

36. Because I would feel bad about myself if I didn’t buy sustainable food. *(Introjected Regulation)*
    - Strongly disagree
    - Disagree
    - Neutral
    - Agree
    - Strongly agree

37. Because it is fun and interesting to find sustainable food products in the shop. *(Intrinsic Motivation)*
    - Strongly disagree
    - Disagree
    - Neutral
    - Agree
    - Strongly agree

38. Because others like me better when I am sustainable aware. *(External Regulation)*
    - Strongly disagree
    - Disagree
    - Neutral
    - Agree
    - Strongly agree

39. Because I'd be afraid of the consequences if I'm not buying sustainable food products. *(Introjected Regulation)*
    - Strongly disagree
    - Disagree
    - Neutral
    - Agree
    - Strongly agree

40. Because it helps my image buying sustainable food. *(External Regulation)*
    - Strongly disagree
    - Disagree
    - Neutral
    - Agree
    - Strongly agree

41. Because it is personally important to me to buy sustainable food. *(Identified Regulation)*
    - Strongly disagree
    - Disagree
    - Neutral
    - Agree
    - Strongly agree

42. Because I feel pressured to adhere to sustainable food products. *(Introjected Regulation)*
    - Strongly disagree
    - Disagree
    - Neutral
    - Agree
    - Strongly agree

43. Because I have a strong value for sustainability. *(Identified Regulation)*
    - Strongly disagree
    - Disagree
    - Neutral
    - Agree
    - Strongly agree

44. For the pleasure of discovering and mastering new things I try to buy sustainable food. *(Intrinsic Motivation)*
    - Strongly disagree
    - Disagree

- Neutral
- Agree
- Strongly agree

45. Because I want others to see me as someone, who takes care of him-/herself. *(External Regulation)*
    - Strongly disagree
    - Disagree
    - Neutral
    - Agree
    - Strongly agree

Section 8 – Additional drivers

How important are the following aspects for you when buying a grocery item?

46. The taste of the product?
    - Most important
    - Important
    - Neutral
    - Less important
    - Not important at all

47. The price of the product?
    - Most important
    - Important
    - Neutral
    - Less important
    - Not important at all

48. The quality of the product?
    - Most important
    - Important
    - Neutral
    - Less important
    - Not important at all

49. The convenience of the product?
    - Most important
    - Important
    - Neutral
    - Less important
    - Not important at all

50. The healthiness of the product?
    - Most important
    - Important

- Neutral
- Less important
- Not important at all

51. The sustainability of the product?
    - Most important
    - Important
    - Neutral
    - Less important
    - Not important at all
